# RUCS: rapid identification of PCR primers for unique core sequences

**DOI:** 10.1093/bioinformatics/btx526

**Published:** 2017-08-30

**Authors:** Martin Christen Frølund Thomsen, Henrik Hasman, Henrik Westh, Hülya Kaya, Ole Lund

**Affiliations:** 1 Department of Bio and Health Informatics, Technical University of Denmark, Lyngby, Denmark; 2 Department of Microbiology and Infection Control, Statens Serum Institut, Copenhagen, Denmark; 3 Department of Clinical Microbiology, Hvidovre Hospital, Copenhagen, Denmark; 4 Institute of Clinical Medicine, University of Copenhagen, Copenhagen, Denmark

## Abstract

**Motivation:**

Designing PCR primers to target a specific selection of whole genome sequenced strains can be a long, arduous and sometimes impractical task. Such tasks would benefit greatly from an automated tool to both identify unique targets, and to validate the vast number of potential primer pairs for the targets *in silico*.

**Results:**

Here we present RUCS, a program that will find PCR primer pairs and probes for the unique core sequences of a positive genome dataset complement to a negative genome dataset. The resulting primer pairs and probes are in addition to simple selection also validated through a complex *in silico* PCR simulation. We compared our method, which identifies the unique core sequences, against an existing tool called ssGeneFinder, and found that our method was 6.5–20 times more sensitive. We used RUCS to design primer pairs that would target a set of genomes known to contain the *mcr-1* colistin resistance gene. Three of the predicted pairs were chosen for experimental validation using PCR and gel electrophoresis. All three pairs successfully produced an amplicon with the target length for the samples containing *mcr-1* and no amplification products were produced for the negative samples. The novel methods presented in this manuscript can reduce the time needed to identify target sequences, and provide a quick virtual PCR validation to eliminate time wasted on ambiguously binding primers.

**Availability and implementation:**

Source code is freely available on https://bitbucket.org/genomicepidemiology/rucs. Web service is freely available on https://cge.cbs.dtu.dk/services/RUCS.

**Supplementary information:**

Supplementary data are available at *Bioinformatics* online.

## 1 Introduction

Polymerase chain reaction (PCR) is one of the most important scientific advances in molecular biology. It is an inexpensive technology for copying a specific sequence of DNA. PCR is an indispensable tool for medical, forensic and research applications. PCR is used for detection and identification of infectious disease agents and for typing and characterization of virulence and resistance genes.

It can be a laborious and often iterative process to design and find primers that only produce the expected amplicons in a PCR reaction. The genome the primers are to bind to can be huge, and the primers may bind to DNA even if the match is not perfect. This unspecific priming can lead to problems with false positive results when PCR is used for detection purposes, or create wrong products for DNA amplification.

Several bioinformatics tools able to help identifying good primer candidates and reduce the amount of false positive results already exist.

Among these tools, the most prominent are ssGeneFinder, Primer3 and PrimerBLAST (Ho *et al.*, 2012; Untergasser *et al.*, 2012; Ye *et al.*, 2012). ssGeneFinder utilizes the BLAST method to find DNA sequences which are unique to a given dataset (Camacho *et al.*, 2009). Primer3 provides a tool for predicting suitable PCR primers for a given DNA sequence. PrimerBLAST expands the usefulness of Primer3 by also allowing for exclusion of primers matching a given database.

Combining these tools provides a possibility to find PCR primer candidates, but the process is not optimal and requires several manual steps to achieve relevant primer candidates. In addition, the ssGeneFinder tool is very specific and will thus miss small unique sequences, like single nucleotide polymorphisms (SNPs) or small insert/deletion.

We have not been able to find any tools that predict if a PCR reaction will lead to problems with unspecific priming, and which work directly on draft genomes. This means that it is often necessary to employ an iterative process with different primer pairs in the lab before a suitable pair is found. The closest tool we could find was FastPCR, but we were not able to make this tool work with draft genomes (Kalendar *et al.*, 2017).

Here we present two novel methods to ease the burden of PCR primer design. The first method (Method 1) is a fast and sensitive method for identifying unique core sequences for a given dataset of positive and negative genomes. The second method (Method 2) combines the primer pair prediction of Primer3, with a novel *in silico* PCR product validation method, which predicts amplicons for PCR primer pairs against a positive and a negative set of references. The results of Primer3 and the PCR validation are then used to sort the pairs according to their PCR performance.

## 2 Materials and methods

### 2.1 Datasets and samples

Two separate studies were undertaken to test the methods described in this manuscript and compare them to existing methods.

The dataset described by Ho *et al.* in relation to validating the ssGeneFinder software (Ho *et al.*, 2012) was used for comparing Method 1 to ssGeneFinder. This dataset contained 9 *Escherichia coli* draft genomes in the positive dataset, and 94 *Escherichia coli*, one *Escherichia albertii*, one *Escherichia fergusonii*, ten *Shigella* and 39 *Salmonella enterica* draft genomes in the negative dataset.

The second study was an *in vitro* laboratory PCR validation of three primer pairs predicted by combining Method 1 and Method 2 on a separate dataset.

The dataset used for study two consisted of 25 *Escherichia coli* (*EC*) samples, which had also previously been subjected to whole genome sequencing: six previously published *EC* samples containing the *mcr-1* gene conferring transferable colistin resistance (Hasman *et al.*, 2015) and 19 previously published *EC* samples lacking *mcr-1* gene (Roer *et al.*, 2017). In addition, three *EC* samples, which were not included in the dataset, were included in the PCR evaluation. One sample with the *mcr-1* gene was added as a positive control, one sample without the *mcr-1* gene was added as a negative control (Roer *et al.*, 2017), and one sample with the *mcr-2* gene, which has 76.7% nucleotide identity to *mcr-1* (Xavier *et al.*, 2016), was added as a negative control for the specificity of the method.

### 2.2 Method 1: Identification of unique core sequences

**Fig. 1:**
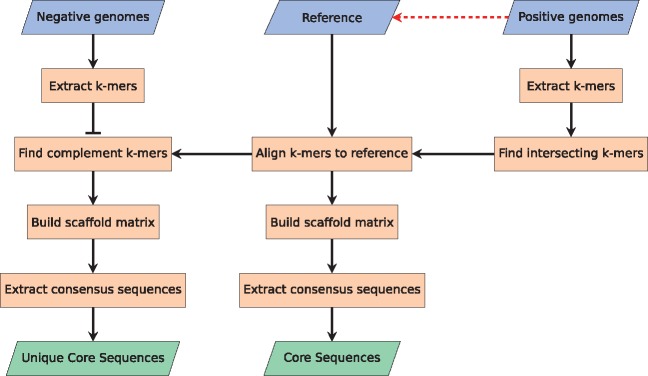
Flowchart depicting the process of Method 1. The three blue parallelogram boxes in the top indicate user input, the two green parallelogram boxes in the bottom indicate output, and the red dashed arrow indicates that the reference can be automatically chosen among the positive genomes. The method is based on a k-mer approach (converting DNA sequences to overlapping oligo nucleotide of length k). This approach is very fast and very accurate, but it leaves one with a result in the unfriendly k-mer space which is not that useful. To solve that, the k-mers are combined to form longer sequences. First, the k-mers are aligned to the reference. Second, the aligned k-mer sequences are used to build a scaffold matrix. Last, a consensus sequence is inferred from the scaffold matrix (Color version of this figure is available at *Bioinformatics* online.)

The first method performs the task of identifying sequences unique to the given dataset. Figure 1 illustrates the workflow of the first part.

The method takes two inputs, a set of genomes containing some common genetic feature (referred to as the positive dataset), and a set of genomes that does not contain this genetic feature (referred to as the negative dataset). The genomes provided can be either draft genomes or closed genomes, and the format must be FASTA. To convert the raw sequencing reads to draft genomes, we recommend using a good assembler. The better the quality of the assemblies, the better the results from this method.

The method also has the option of selecting a reference genome, this genome is used solely to map the k-mer, and as such it should be part of the positive set of genomes. The default reference is the first of the positive genomes, but for the best results, we recommend using the best assembled positive genome as the reference.

#### *2.2.1* Extraction of k-mers

A k-mer is an oligonucleotide consisting of k bases. A k-mer extraction refers to the extraction of all overlapping and non-overlapping k-mers for a given sequence. The default k-mer size is 20, which was found to give a good balance between specificity and sensitivity. If the k-mer size is too short, too many k-mers will be present in the negative dataset, and if the k-mer size is too long, then the positive dataset will have too few k-mers in common. Both the k-mers on the forward and the reverse strand are used.

#### *2.2.2* Finding intersecting k-mers

The k-mers across all the positive samples are compared, and only those common to all will be kept as the positive core k-mers.

#### *2.2.3* Aligning k-mers to the reference

The k-mers are mapped to the reference genome through BWA alignment (Li and Durbin, 2009). Only the forward strand of the reference and only perfect matches are considered, and since all k-mers are present in the reference, either the k-mer or its reverse complement is bound to be present.

#### *2.2.4* Finding complementing k-mers

All positive core k-mers that are found in any of the negative samples are removed from the resulting unique core k-mers.

#### *2.2.5* Building scaffold matrices

The pre-aligned k-mers are piled up and a position-wise stack count is computed. Basically, this means that a count is logged for each position in the scaffolds, based on the number of overlapping k-mers. This count is an integer between 0 and the k-mer size, and it can later be used to estimate how likely a given position is to be unique.

#### *2.2.6* Extracting consensus sequences

The consensus sequence for each segment of overlapping k-mers is extracted and stored as a FASTA file (referred to as contigs). Using the contigs, the method generates a scaffold FASTA file mimicking the reference, but where all bases not found in the contigs are stored as n's. In addition to the normal scaffolds, the method creates another FASTA file (referred to as dissected scaffolds), where all n-stretches longer than x bases are cut out, and the resulting fragments stored as separate dissected scaffolds. The default value of *x* is 1000, which fits well with the maximum length one would want in classic PCR (x is modified according to the user set max amplicon length). This dissected scaffold format is important for the utilization of the methods sensitivity, as just a single SNP in the positive core sequences causes the sequence to be split in two contigs. By providing the dissected scaffold to the Primer3 software, in the next method, the software is able to make suggestions across the contigs (Untergasser *et al.*, 2012).

### 2.3 Method 2: PCR *in silico* simulation

**Fig. 2:**
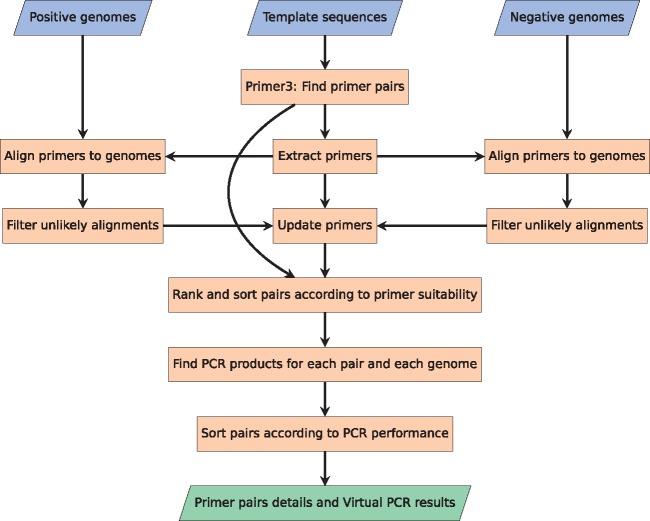
Flowchart depicting the process of Method 2. As with figure 1, the three blue parallelogram boxes in the top are input and the green parallelogram box in the bottom is output. To find potential primer pairs, the well-used Primer3 software package is utilized on the template sequences. The identified primers are then aligned to all the provided genomes, and potential alignments to the genomes are stored with the primers. The primers are then ranked and sorted according to their suitability. In the end, each pair is tested for PCR products against all references, and afterwards sorted according to their PCR performance (Color version of this figure is available at *Bioinformatics* online.)

The second method finds potential PCR primer pair candidates in the provided template sequences and simulates an *in silico* PCR reaction for all primer pair candidates against all samples, both positive and negative, in the dataset. When using Method 1 in combination with Method 2, the template sequences are the dissected scaffold sequences. Figure 2 illustrates the workflow of this method. Just as for Method 1, Method 2 also needs a positive and negative set of genomes in FASTA format as input.

#### *2.3.1* Finding potential primer pairs (Primer3 software)

The method starts by running the provided template sequences through the Primer3 software (via the Python package). The Primer3 software locates around 10 000 primer pair candidates for a sequence at a time. From these results, the primer sequences (and probes) are extracted to a file. The Primer3 software supports identification of hybridization probes, this option is also integrated in Method 2, so all mentions of primer pairs also imply a probe, when this option is selected.

#### *2.3.2* Aligning primers to the genomes

All the primer and probe sequences are BLASTed against all of the positive and negative genomes with loose BLAST settings to find potential binding location (Camacho *et al.*, 2009).

#### *2.3.3* Filtering unlikely binding locations

By leaving the BLAST settings unconstrained, improbable alignments are inevitable. To get rid of these unlikely binding location, the alignments are analyzed, graded and filtered according to the default grading scheme shown in Table 1. The filtering process happens in two steps. First filtering step compares the sequence similarity of the alignment, and all matches with less than 60% similarity get the grade 0 and are excluded from filtering step 2. After this first filtering step, all the alignments undergo a hetero dimer thermodynamic analysis using the Primer3 software. A melting temperature (*T*_m_) prediction above 0 °C gets grade 1, a *T*_m_ prediction above 47 °C gets grade 2, a *T*_m_ prediction within 5 °C of the target temperature gets grade 3 and a *T*_m_ prediction within 1 °C of the target temperature gets grade 4. All alignments below the default threshold grade (2) are considered unlikely binding locations, and are removed from the alignments.

**Table 1.** Hetero dimer melting temperature (*T*_m_) grading scheme

Grade	Description	Rule
0	No priming	Alignment similarity < 0.6 or *T*_m_ < = 0 °C
1	Unlikely priming	Alignment similarity > 0.6 and *T*_m_ < 47 °C
2	Potential priming	*T*_m_ > 47 °C
3	Probable priming	*T*_m_ = 60 °C ± 5 °C
4	Definite priming	*T*_m_ = 60 °C ± 1 °C

*Note*: The 60 °C is the default annealing temperature. These threshold values can be modified in the settings file.

#### *2.3.4* Identification of probable PCR products

With the primers' priming potential computed, each pair is analyzed with regards to the PCR products produced for each genome in the datasets.

#### *2.3.5* Identification of best primer pairs

The primer pairs are ranked according to their suitability. A perfect pair will only produce the target product size (or produce an amplicon with a binding probe sequence for qPCR) for all the positive samples, and no product can be present in any of the negative samples. The further away from perfect, the worse the ranking. The top picks are presented first.

To help the user select the best pair(s), the second method will perform a BLASTx search against the RefSeq protein database to annotate the resulting amplicons of the pairs with useful information (Agarwala *et al.*, 2016; Camacho *et al.*, 2009).

### 2.4 The tool

The tool containing the two methods presented in this manuscript has been dubbed **R**apid identification of PCR primers for **U**nique **C**ore **S**equences (RUCS). The source code for RUCS is freely available. The tool was implemented in Python, and has several dependencies such as BLAST and BWA. To make the tool easy to install, the tool has been installed in a Docker container, and the image can be found at Docker Hub.

To make the use of the tool even more convenient, an online webserver has been set up to run the service. However, due to server constraints, the webserver is limited as to how many genomes and template sequences that can be processed per submission.

The RUCS API provides six different entry points, which are all available from the web service:

1. Combine the two methods described in this manuscript for a full run to design PCR primer pairs for the unique cores sequences of a positive dataset versus a negative dataset (Method 1 + Method 2).
2. Find sequences which are unique to a dataset of positive samples compared to a dataset of negative samples (Method 1).
3. Identify PCR primer pairs for a given set of sequences (Method 2).
4. Run PCR *in silico*, for a given set of primer pairs against a given set of references (Method 2 without primer design and sorting).
5. Annotate a given set of sequences for protein annotations from the NCBI RefSeq database (BLAST).
6. Show PCR statistics for a given primer set to a given template (Primer3).

The parentheses next to each entry point describe which method or third party software is responsible for the results.

For each run, a statistics log is generated containing information such as: processing time for each part of the program, sequence analysis details, Primer3 scanning details, primer binding prediction analysis details and PCR product analysis details.

## 3 Results

To test and validate the two methods presented in this manuscript, two separate studies were conducted.

### 3.1 Summary of Study 1: comparison to ssGeneFinder

This first study, which compared Method 1 to ssGeneFinder, showed that Method 1 was able to identify 6.5–20 times more unique sequence data and 2.4 times more eligible primer binding sites compared to ssGeneFinder, with only a marginal increase in compute time (∼38%). For the full study, see Supplementary File S1.

### 3.2 Summary of Study 2: PCR validation

The second study was an *in vitro* laboratory PCR validation of three primer pairs predicted by combining Method 1 and Method 2 on an *EC* dataset where the positive genomes were known to contain the *mcr-1* gene.

Running the dataset of 6 *EC* samples containing the *mcr-1* gene and 19 *EC* samples known not to have the mcr-1 gene through Method 1 produced a single dissected scaffold above the threshold of 300 bp and containing the *mcr-1* gene. Running this dissected scaffold through Method 2 produced 9986 eligible PCR primer pair candidates of which we randomly chose three pairs for further *in vitro* PCR validation (Table 2).

**Table 2.** List of the three PCR Primer Pairs chosen for *in vitro* PCR validation

Name	Amplicon size	Forward primer	Reverse primer
1-mcr1	408	5′- ATTATCCGACTTGGGGCAAGG -3′	5′- CGCACGATGTGACATTGCTAA -3′
2-mcr-1	382	5′- ACGCCAGTGTGTGAAGGTAAT -3′	5′- CGGTGCGGTCTTTGACTTTG -3′
3-mcr-1	306	5′- CTGACACTTATGGCACGGTCT -3′	5′- TCGGATTGACATAGCTACGCA -3′

*Note*: Size is provided in bases.

For the *in vitro* PCR validation experiment, we added three additional *EC* samples, one extra *mcr-1* positive strain, one extra *mcr-1* negative strain and one strain carrying the *mcr-2* gene. The 28 *EC* samples were then combined with all combinations of the PCR primer pairs, giving a total of 84 PCR reactions.

The results of the 84 PCR reactions showed that the output from running a combination of Method 1 and Method 2 on dataset 2 is able to predict useful PCR primer pairs. In this case, all three primer pairs were able to identify all strains containing the *mcr-1* gene, while they did not produce any visible PCR amplification from the *mcr-1* negative strains or from the strain carrying *mcr-2* gene. See Supplementary File S2 for more details.

## 4 Discussions

Here we present a sensitive and specific method for finding primers that target regions uniquely shared among all samples in a positive genome dataset but not found in any samples from a negative set. It is validated both by an *in silico* benchmarking set and by experimental analysis. A wise selection of negative samples is imperative for avoiding primer pairs that target undesired regions. The combination of Method 1 and 2 selectively finds primer pairs, which do not produce an amplicon for any of the negative samples included in the training data. This, however, does not ensure that the identified pairs will not bind to negative samples excluded from the training data.

As the ssGeneFinder tool utilizes BLAST to identify similar DNA targets, it is better suited for finding targets with some degree of sequence variation among the positive samples, whereas Method 1 is more stringent and only reports exact sequence matches. Also for Method 1, those sequence matches need to be present in all the positive samples, whereas ssGeneFinder allows for a fraction of the samples not to have the target. Even though we showed that Method 1 is more sensitive than ssGeneFinder, the opposite might also be true in some cases. Thus, it is important to know what one is looking for, before choosing any single method.

## 5 Conclusion

Here we presented two novel methods which can be combined to create a tool for identifying highly specific PCR primer pairs that bind uniquely to a positive dataset, and the tool does not make any PCR product for the negative dataset. We have compared Method 1 to a similar tool called ssGeneFinder and found that Method 1 is more sensitive and finds more potential binding sites for uniquely binding PCR primer pairs. We have also validated three of the predicted primer pairs *in vitro* using classic PCR. The PCR validation showed that all three primer pairs were able to identify the presence of the *mcr-1* colistin resistance gene.

The software source code is publicly available online at https://bitbucket.org/genomicepidemiology/rucs. In addition, a pre-installed Docker image, for easy installation across all platforms, can be found at https://hub.docker.com/r/genomicepidemiology/rucs/, and a web service implementing a limited version of the software can be found at https://cge.cbs.dtu.dk/services/RUCS.

## Supplementary Material

Supplementary Data
