# Supplementary material for: RUCS: rapid identification of PCR primers for unique core sequences
Source: Bioinformatics. 2017 Aug 30;33(24):3917–21. doi: 10.1093/bioinformatics/btx526 (PMC5860091; doi:10.1093/bioinformatics/btx526)
Supplement: Supplementary Data [file btx526_supp.zip › btx526-suppl_data/file1.docx]

**Background**

This manuscript describes a new method (Method 1) for identifying unique sequences given a positive and a negative dataset. This study compares Method 1 to an already existing tool called ssGeneFinder (ssGF), which uses a more conservative approach to finding unique sequences.

**Materials and Methods**

The dataset used for this comparison is obtained from the original manuscript of ssGF, and the content have been shortly described in this manuscript.

For this study, we used the nine positive draft genomes as the positive dataset, and the 145 negative draft genomes as the negative dataset. For reference we chose the same as in the ssGF manuscript, T0005Escherichia_coli_TY_2482_20110606_upload2ncbi.fas. Mostly the default settings were used, though a few modifications were made: The PRIMER_PRODUCT_SIZE_RANGE was changed to 50-1000 bases, the PRIMER_NUM_RETURN was upped to 50000, and the threshold_grade was changed to 1. These three modifications were made to optimise Method 1 towards identifying more unique binding primers.

For the setup of the second method described in this manuscript (Method 2), the template was the same as the default for a full run, unique_core_sequences.disscafs.fa, but the seq_selection was set to ['3'] so that Method 2 would only target the dissected scaffold of interest.

**Results**

Running the dataset through Method 1 produced 94618 bases worth of unique core sequences, which is a 20-fold increase compared to the 4644 bases reported by ssGF. Analysing the k-mer depth provided in the unique_core_sequences.aux.tsv file, we see that of the 94618 bases, 64237 are covered by 10 or more unique k-mers, and 30047 bases are fully covered by the maximum amount of k-mers (20). So even with the most conservative criteria, Method 1 provides a 6.5-fold increase in unique bases compared to the reported results from ssGF. A comparison of the results can be found in Table 1.

Though Method 1 outperformed ssGF on the sheer amount of unique data identified, more unique sequence material do not necessarily translate into more unique primer binding sites. To investigate how much the extra data impact the amount of unique primer binding sites, a single dissected scaffold with a length of 4599 bases was selected for further analysis. This scaffold was chosen since it contained the second greatest amount data from ssGF's unique.fasta file. Note, the biggest ssGF fragment is identical to the corresponding fragment found by Method 1, which makes it uninteresting for comparison. On the chosen scaffold, located on contig 65 in the reference, ssGF identified two sequences covering 1203 bases, and Method 1 identified 10 sequences covering 3175 bases.

Superimposing the ssGF results (yellow marking) on top of the dissected scaffolds, showed the sensitivity of Method 1 compared to ssGF (See Figure 1).

The dissected scaffold was run through Method 2, which identifies PCR primer pairs for a given sequence and validate the predictions using a PCR *in silico* simulation. We extracted the uniquely binding primers from the results, and marked their locations on Figure 1 by highlighting the participating bases with red colour.

It is clear from Figure 1 that there are suitable binding sites, which are only picked up by Method 1. 59% of the unique-primer-covered bases are located outside of the sequences found by ssGF (1248 out of 2105 bases). It is also partly confirmed that not all the bases predicted by Method 1 provide a unique binding site for primers, though just above 66% of the predicted bases in the dissected scaffold was predicted to participate in binding unique primers (2105 out of 3175 bases). This observation emphasize the importance for the *in silico* PCR, to verify the suitability of the primer pairs, and avoid wasting time testing compromised primer pairs *in vitro*.

On a side note, these results show the advantage of using the dissected scaffolds over the contigs, since Method 1 would miss out on several viable primer pairs if it ran on the contigs.

In regards to the compute time, ssGF states in the corresponding paper that it took 24 minutes to analyse the data. Method 1 ran on a MacBook Pro from 2013 using 1 core, and required 33 minutes to analyse the data. The methods operate on two different operative systems, which ruled out a clean comparison of the methods resource consumptions.

**Conclusion**

This study showed that Method 1 identified 6.5-20 times more unique data than ssGF, and of the bases covered by uniquely binding primers, predicted by Method 2, only 41% of the bases were covered by ssGF. In regards to resources, ssGF is around 38% faster than Method 1.

Disclaimer: This study did not show the result of an exhaustive test of potential primer-binding sites, so there could be more viable primer-binding sites in the plot. The purpose of this study was to investigate if Method 1 could predict more unique sequences material compared to ssGF, and whether the additional material also translates into more viable PCR primer-binding sites. This study clearly demonstrated that there are sequences outside of the regions identified by ssGF, which are viable options for uniquely binding PCR primer pairs.

***Table 1 - Sequence analysis results****: Size reports the number of bases across all sequences, contigs report the number of sequences in the fasta output.*

| **Name** | **Size** | **Contigs** | **N50** |
| --- | --- | --- | --- |
| ssGF reference | 5291054 | 451 | 53266 |
| Method 1 dissected scaffolds | 285214 | 380 | 3425 |
| Method 1 contigs | 94618 | 1458 | 64 |
| ssGF targets ref (TARGETusidAll.fasta) | 4494 | 11 | 1054 |
| ssGF targets all (unique.fasta) | 4644 | 13 | 1054 |

>dissected_scaffold_on_contig_65

CC**GTTCTGATGCTATGCTGATAGCGGTTAATGATTTCTC**nnnnnnnnnnnnnnnnnnnnnnnnnnnnnnnnnnnnnnnnnnnnnnnnnnnnnnnnnnnnnnnnnnnnnnnnnnnnnnnnnnnnnnnnnnnnnnnnnnnnnnnnnnnnnnnnnnnnnnnnnnnnnnnnnnnnnnnnnnnnnnnnnnnnnnnnnnnnnnnnnnnnnnnnnnnnnnnnnnnnnnnnnnnnnnnnnnnnnnnnnnnnnnnnnnnnnnnnnnnnnnnnnnnnnnnnnnnnnnnnnnnnnnnnnnnnnnnnnnnnnnnnnnnnnnnnnnnnnnnnnnnnnnnnnnnnnnnnnnnnnnnnnnnnnnnnnnnnnnnnnnnnnnnnnnnnnnnnnnnnnnnnnnnnnnnnnnnnnnnnnnnnnnnnnnnnnnnnnnnnnnnnnnnnnnnnnnnnnnnnnnnnnnnnnnnnnnnnnnnnnnnnnnnnnnnnnnnnnnnnnnnnnnnnnnnnnnnnnnnnnnnnnnnnnnnnnnnnnnnnnnnnnnnnnnnnnnnnnnnnnnnnnnnnnnnnnnnnnnnnnnnnnnnnnnnnnnnnnnnnnnnnnnnnnnnnnnnnnnnnnnnnnnnnnnnnnnnnnnnnnnnnnnnnnnnnnnnnnnnnnnnnnnnnnnnnnnnnnnnnnnnnnnnnnnnnnnnnnnnnnnnnnnnnnnnnnnnnnnnnnnnnnnnnnnnnnnnnnnnnnnnnnnnnnnnnnnnnnnnnnnnnnnnnnnnnnnnnnnnnnnnnnnnnnnnnnnnnnnGG**CGCAAATACAGTAACGAATCTCTTTGCCCTTTTTGTTTTTCT**nnnnnnnnnnnnnnnnnnnnnnnnnnnnnnnnnnnnnnnn**AACCAATGAATACAGAAAACCCACAATCCTTTATTCTTAAGGCTGTAAAAGAACTGGCAGCTATTAGCGAGGAATCAGTTATAAACACATCGG**CGCTTTGCC**GCCTTTTAGAAATTGATGCAAATAATGTGC**GCCAGCGTGTCTTTCAGACTGGGTGCAG**CACTTTCGAAGCAATTCAATACTATTG**CTCTAAAAAACAATAAT**AAACAACAATCATAATACAGGGAAACAATTAGCATGTTAGAACTTAACGTCAGCCGCTTATCAAACGTTGTCACCTTCCAGGTTGATCGACCATCATTAGAGAAAGCAAAAGCAGAAGTAGAAAAACTTCGTAAACAAATGGCGGCGGTGAAAGATATAGAATTGCGTGTTAAGACACATAAACAATCAACTTCTAAAGCAAAAAAAGACGTAGACGATATTGCTAAAAAGC**AAGCACAGGCAGATAAAGCCAATGCTAAAGCACAATTAGnnnnnCAACGT**GTAGTTGCAAGAGAACAAAAAG**CCCTGGCAGCACGC**AAAGAGAAGGCAGACTTAAAACTGTTAGACGTTGGAT**CAAGCATTAGTGCATTGCACCGCC**TTTCTGTAGCGGAACAATATAAAGCTATTG**CACAGGCTAGAGAAATTGCCCT**CCAATATGAAAGAGGAGCTATTAGTCTG**GCACGTATGAATAGCCAAAnnnnnnnnnnnnnnnnnnnnnn**ACGTAAGATTAATGGCAATCGTAACG**CTCACGCTAAAGCCTATGCACCTGTTAAGGGTAGCGGGAGTATTGGCGGCGGTGCTGCGGCTGTTATGCTGGGTGGCTTAGGCGCTGGC**GCTACGTATATGGCTATGAGCAAAGCCTCTGAATTTGTTACTAACA**GCTTTGCGAATGCTGAAACGCTGGGTGAATTGTATAGCCGTGCGAAGCTGGGCGGT**GTAGACGTTAACCAGATGAACAATATTGAGCAATGGG**CCTACAAAAACGGCGTTGATTCAATGATGGG**GGATCAAGGTAAACGTAAGTATCTCGATCAGATGAAAGATGT**GCGTGAGCGTG**CAACCAAAAGCTATGATGAGGCTGAATATGTAGTCGATAAAA**AAACAGGCAAAGGTGAATGGAAGGGCGGCG**ATAGCGGCATCAATACTTTAATGAATGAAGGATTCTTAACTAAAAAGGATCTAAAAGACTTTGCTGATAATCC**GGCTGG**TCTGGTTAGTAAAGCAGTTC**AGGG**AATGGTGAAGAAAGGCTATTCAGATGCACAAATCG**GTAGTCGTCTGGAAGATCTGGCTGATG**ATTTGATGCTCACCTCTAAATACTGGAC**ACGCTCC**GCTAAAGAAGTGGAACAATCAG**CACGAGAACAACGAGAATCAGGGCGCTGGGTAACACAGGCA**CAACAGGAAAGCGTTATTAAATTCCGTGAACTGAATAATGCACTTAGTGG**GCTTTCTGACTCACAAGGGATCGC**CTTTGTTGATGGCTTTATGAAATCTCTTGATCCGAAAGTGATTGAAGAGTTTAAAAAGAGCATGGTTG**CAATG**CTGCCAATGTTTACCAAATTAGGCG**AGGCTATCGGCGGATTGGTTAAC**GCCGTAATGAAAACAATCAACTGGC**TGATGAAAAACGACGAGAAGACGGAAGCTA**TTCAGAAGAATTTAGGGGATG**CACCG**CCACTATCTAACGAAGGAATGAAGCAGAATCTTTCTAATCTCACTCCTGATCAATATAAAGGCG**CTGGCACC**GGAACAACTAAGCCAGATAACAGCAATTCCCTGGTTAACACTATTAAAAGCTGGTTTATGCCAGAAGAAACGGTAGGTGGGGCGCAGGCTGTAAATCAATACAGTCTCGAAGGACAAAACATTGCAAATCTGAAACAGAGTGCAGCACAACAAGCTTTCAAGGCTCCTTCTTACACCTTTGCACCTGTTATTAACTTCAATCCAGAATTACAAGTAAATGCAGAAGTGCCGCTAACTATCGAATCAGACACCGGACGATTGAGCGAATTTATCGACTTCAAATCAAAGGCATCCTCCGCTGAGTTTAGCAAGCTATTAACGCTGGGTGTTATGTCAGGCGGGTCAACCTATTAATCATCACTGTGGGGCTGTTTAGCCCCCTTAAATAAGGATTACAAATTATGGCAATGGGTCCGTTAACCATTGGTCGCCCGACTGAGGCGAAAATCAACAGCACAAGCAACGATAGCAACGGTAGAACAACCAAAGGGGGTAACGGCTTTGCGATCATTACCTCTAATCTCGGCAGTGGTGCAAATGCTGCTTACAATGACTACCAGGCATTATCATTTGATAGCGTGGAATCGACGAAAGTTAGCAGAAATGCGGACGTTACAAGCTATGCCGTCGAATCAGGATCAGAAGTTAGTGATCATGTTCAAATACGGAATAACAAGTTCACATTACAAGGGCGTATCTCTGAAACCGTATTGAAGCTCAATCCTGATATGATTAAGAATGCTGGAATTAACGGCAATCGACGTATGCTTATGCTTGAATACCTAAATCAGTTGATGGACAGTCGCCAACCTTTCCTACTTGTCACTGAGTTAAAAAATTATGACAACGTTGTGTTAGTTGGCATGTCTTACGAAGAAGAAGCAAGCGAAAGCCTATTGTTTACTCTTGATTTTGAGCAAATCCGTCTTGTCTCTAAAGCAACTACAAGCGCAATTGCGGTAAAAACAGCACCAAATAAATCTGTAGGAGGCCAGGTTAAAATGCAGGTAAATACAACAGAACAGAAAAATCAAAAAAGCCCAGGGCAAGACGTTGTAACGCCAGTATTTAAACAATAGCCTTAAACGCCTATGAAACGCTCTGTAACGAGATAATCATAAAAGAGGCATCAGTCTATATTGAAGCCTCTTTATTACGTCTATAGCGTTGTTTTACG**CCTTAAnnnnnnnnnnnnnnnnnnnnnnnnnnnnnnnnnnnnnnnnnnnnnnnnnnnnnnnnnnATATATTTTTTAAGCTCTATTGGA**GAAGCTGCATTTTTGCTATATGT**GCGGAATGnnnnnnnnnnnnnnnnnnnnnnnnnnnnnnnnnnnnnnnnnnnnnnnnnnnnnnnnnnnnnnnnnnnnnnnnnnnnnnnnnnnnnnnnnnnnnnnnnnnnnnnnnnnnnnnnnnnnnnnnnnnnnnnnnnnnnnnnnnnnnnnnnnnnnnnnnnnnnnnnnnnnnnnnnnnnnnnnnAGCGTTTACCGTCAGCACGTTCTGTAATGCTGGCGTGATnnnnnnnnnnnnnnnnnnnnnnnnnnnnnnnnnnnnnnnnCAC**CAACGGAGTGATAAGGTTATG**CACAGGCACAACACGnnnnnnnnnnnnnnnnnnnnnnnnnnnnnnnnnnnnnnnnnnnnnnnnnnnnnnnnnnnnnnnnnnnnnnnnnnnnnnnnnnnnnnnnnnnnnnnnnnnnnnnnnnnnnnnnnnnnnnnnnnnnnnnnnnnnnnnnnnnnnnnnnnnnnnnnnnnnnnnnnnnnnnnnnnnnnnnnnnnnnnnnnnnnnnnnnnnnnnnnnnnnnnnnnnnnnnnnnnnnnnnnnnnnnnnnnnnnnnnnnnnnnnnnnnnnnnnnnnnnnnnnnnnnnnnnnnnnnnnnnnnnnnnnnnnnnnnnnnnnnnnnnCCCATAGCTGG**GCCATTGGACTGATATAATTTGCAATTG**nnnnnnnnnnnnnnnnnnnnnnnnnnnnnnnnnnnnnnnCTGTAACAGTGGTGCGGCTTACATCTTGCAATGCAATAT

***Figure 1 - Dissected scaffold from contig 65:*** *The sequences found by ssGF are marked with yellow background colour, and the bases covered by primer binding sites are marked in red.*
