# Supplementary material for: RUCS: rapid identification of PCR primers for unique core sequences
Source: Bioinformatics. 2017 Aug 30;33(24):3917–21. doi: 10.1093/bioinformatics/btx526 (PMC5860091; doi:10.1093/bioinformatics/btx526)
Supplement: Supplementary Data [file btx526_supp.zip › btx526-suppl_data/file2.docx]

**Background**

This manuscript describes two new methods, Method 1 and Method 2. Method 1 identify sequences unique in a given dataset (positives) compared to another given dataset (negatives). Method 2 finds and validates *in silico* primer pair candidates designed from the resulting sequences of Method 1. The result is a sorted list of primer pairs that target the positive samples specifically, producing no amplicons for the negative sample. Please see the manuscript for further method details. To validate these predictions, this study will analyse, by an *in vitro* approach, three randomly chosen sets of primer pairs predicted by these methods on an *Escherichia coli* (*EC*) data set. For the training/prediction part, we used the six positive genomes and the 19 negative genomes, and for the *in vitro* evaluation, we included the three additional genomes, as described in the manuscript.

**PCR Experiment Setup**

0.2 µM of each primer were combined with the QIAGEN Multiplex PCR Master Kit (QIAGEN, Valencia, CA, USA) in a 25 µL reaction. Amplification was performed with the following settings: 15 min at 94°C, followed by 30 cycles of 30 s at 94°C, 90 s at 60°C, 60 s at 72°C and a final extension of 10 min at 72°C. T. Visualization of the PCR products were performed on 2% E-Gels (Invitrogen, Gran Island, CA, USA).

**Results**

Running Method 1 on the dataset produced only one dissected scaffold above 300bp (2599bp in length). This dissected scaffold contained the two longest unique core sequences with one n in between, and the *mcr-1* gene was located in this sequence. See Table 1 for more details. Running this dissected scaffold through Method 2 produced 9986 PCR primer pairs, which all passed the *in silico* PCR validation included in Method 2. Three of these pairs were chosen for *in vitro* PCR validation (see Table 2). The predicted binding locations can be seen in Figure 1. For the experiment, the 28 *EC* samples (see Table 3) were used with all combinations of the PCR primer pairs, giving a total of 84 PCR reactions. The gel layout for the 84 PCR reactions is described in Table 4.

All 84 PCR reactions went as predicted (see Figure 2 and 3).

**Conclusion**

The results of the 84 PCR reactions showed that the combination of the methods, Method 1 and Method 2, is able to predict useful PCR primer pairs. In this example, all three tested primer pairs performed exactly as intended.

***Table 1 - Sequence Analyses****: The results of Method 1 on the mcr-1 dataset produced three unique core sequences.*

| Fasta file | Sequences | Size in bases | Seqs >300 | Size >300 |
| --- | --- | --- | --- | --- |
| reference.fa | 187 | 5701668 | 185 | 5701296 |
| core_sequences.contigs.fa | 52158 | 2950617 | 415 | 180929 |
| unique_core_sequences.contigs.fa | 3 | 2626 | 1 | 2458 |

***Table 2 - PCR Primer Pairs****: Three primer pairs were chosen from the analysis for testing in vitro using PCR and gel electrophoresis.*

| Name | Size | Forward Primer | Reverse Primer |
| --- | --- | --- | --- |
| 1-mcr1 | 408 | 5'- ATTATCCGACTTGGGGCAAGG -3' | 5'- CGCACGATGTGACATTGCTAA -3' |
| 2-mcr1 | 382 | 5'- ACGCCAGTGTGTGAAGGTAAT -3' | 5'- CGGTGCGGTCTTTGACTTTG -3' |
| 3-mcr1 | 306 | 5'- CTGACACTTATGGCACGGTCT -3' | 5'- TCGGATTGACATAGCTACGCA -3' |

***Table 3 - Sample list****: Overview of the strains used in the in vitro experiment.*

| Sample ID | Sample Name | PUBMED | Run Accession | Used for prediction? |
| --- | --- | --- | --- | --- |
| P1 | 14042624_1_CLC | 26676364 | ERR1399398 | Yes |
| P2 | 0412049521_F168_2844-11-B_CLC | 26676364 | ERR1399397 | Yes |
| P3 | DTU2014_233_0413040864_CLC | 26676364 | ERR1399394 | Yes |
| P4 | E_coli_0412016126_F27_CLC | 26676364 | ERR1399395 | Yes |
| P5 | E_coli_0412044854_F159_CLC | 26676364 | ERR1399396 | Yes |
| P6 | ESBL20150072_CLC | 26676364 | ERR1399393 | Yes |
| P7 | prae-11-102_CLC |  |  | No |
| P8 | mcr-2 strain | 27416987 | LT598652 | No |
| N1 | ESBL20150100_CLC | 28369408 |  | Yes |
| N2 | ESBL20150101_CLC | 28369408 |  | Yes |
| N3 | ESBL20150102_CLC | 28369408 |  | Yes |
| N4 | ESBL20150103_CLC | 28369408 |  | Yes |
| N5 | ESBL20150104_CLC | 28369408 |  | Yes |
| N6 | ESBL20150105_CLC | 28369408 |  | Yes |
| N7 | ESBL20150106_CLC | 28369408 |  | Yes |
| N8 | ESBL20150107_CLC | 28369408 |  | Yes |
| N9 | ESBL20150108_CLC | 28369408 |  | Yes |
| N10 | ESBL20150109_CLC | 28369408 |  | Yes |
| N11 | ESBL20150130_CLC | 28369408 |  | Yes |
| N12 | ESBL20150132_CLC | 28369408 |  | Yes |
| N13 | ESBL20150133_CLC | 28369408 |  | Yes |
| N14 | ESBL20150134_CLC | 28369408 |  | Yes |
| N15 | ESBL20150136_CLC | 28369408 |  | Yes |
| N16 | ESBL20150137_CLC | 28369408 |  | Yes |
| N17 | ESBL20150140_CLC | 28369408 |  | Yes |
| N18 | ESBL20150141_CLC | 28369408 |  | Yes |
| N19 | ESBL20150142_CLC | 28369408 |  | Yes |
| N20 | ESBL20150143_CLC | 28369408 |  | No |

***Table 4 - PCR Gel Setup****: This table shows the position of each sample and primer pair combination. P is for positive and N is for negative samples. The number in front of the Ps and Ns refers to the primer pair used, and the number following the Ps and Ns refers to the sample. The fields containing # are wells containing a ladder.*

|  | **M** | **1** | **2** | **3** | **4** | **5** | **6** | **7** | **8** | **9** | **10** | **11** | **12** | **13** | **14** | **15** | **16** | **17** | **18** | **19** | **20** | **21** | **22** | **23** | **24** | **M** |
| --- | --- | --- | --- | --- | --- | --- | --- | --- | --- | --- | --- | --- | --- | --- | --- | --- | --- | --- | --- | --- | --- | --- | --- | --- | --- | --- |
| **Gel 1 row 1** | # | 1P1 | 1P2 | 1P3 | 1P4 | 1P5 | 1P6 | 1P7 | 1P8 | 2P1 | 2P2 | 2P3 | 2P4 | 2P5 | 2P6 | 2P7 | 2P8 | 3P1 | 3P2 | 3P3 | 3P4 | 3P5 | 3P6 | 3P7 | 3P8 | # |
| **Gel 1 row 2** | # | 1N1 | 1N2 | 1N3 | 1N4 | 1N5 | 1N6 | 1N7 | 1N8 | 1N9 | 1N10 | 1N11 | 1N12 | # | 1N13 | 1N14 | 1N15 | 1N16 | 1N17 | 1N18 | 1N19 | 1N20 | # |  |  |  |
| **Gel 2 row 1** | # | 2N1 | 2N2 | 2N3 | 2N4 | 2N5 | 2N6 | 2N7 | 2N8 | 2N9 | 2N10 | 2N11 | 2N12 | # | 2N13 | 2N14 | 2N15 | 2N16 | 2N17 | 2N18 | 2N19 | 2N20 | # |  |  |  |
| **Gel 2 row 2** | # | 3N1 | 3N2 | 3N3 | 3N4 | 3N5 | 3N6 | 3N7 | 3N8 | 3N9 | 3N10 | 3N11 | 3N12 | # | 3N13 | 3N14 | 3N15 | 3N16 | 3N17 | 3N18 | 3N19 | 3N20 | # |  |  |  |


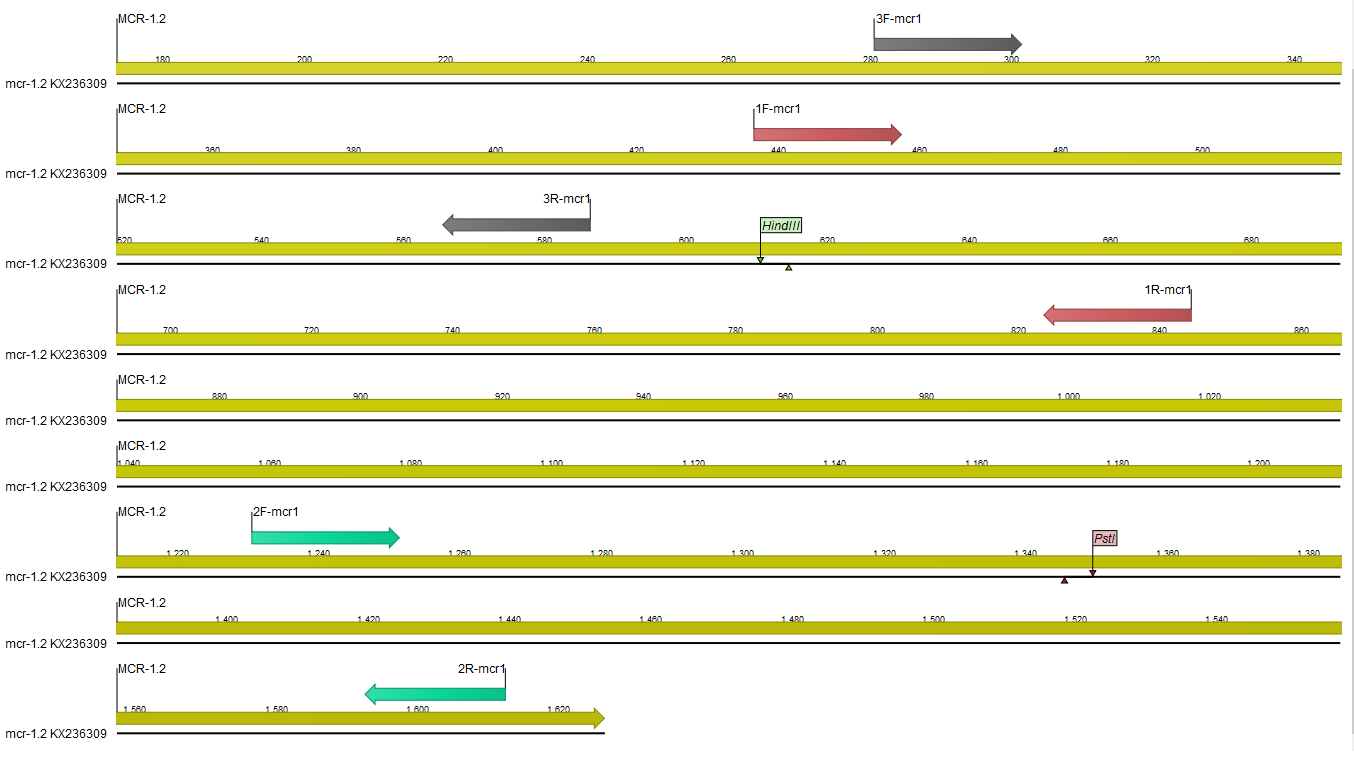


***Figure 1 - Primer Locations****: Diagram showing the binding location of the three primer pairs on the mcr-1.2 template. 1F and 1R are marked in red, 2F and 2R are marked in green, and 3F and 3R are marked in grey. Figure is created by CLC Genomic Workbench 9.5.3 (Qiagen).*


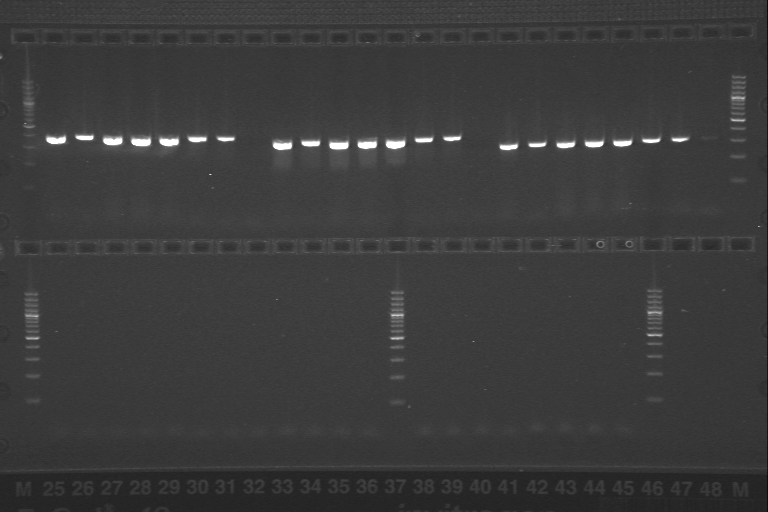


***Figure 2 - Gel 1****: Gel electrophoresis results of for the first gel. The top row contains P1 through P8, first with pair 1, then with pair 2 and pair 3. All, except P8, shows the target band length. The bottom row contains N1 through N20 mixed with pair 1. None of the negative samples show any bands.*


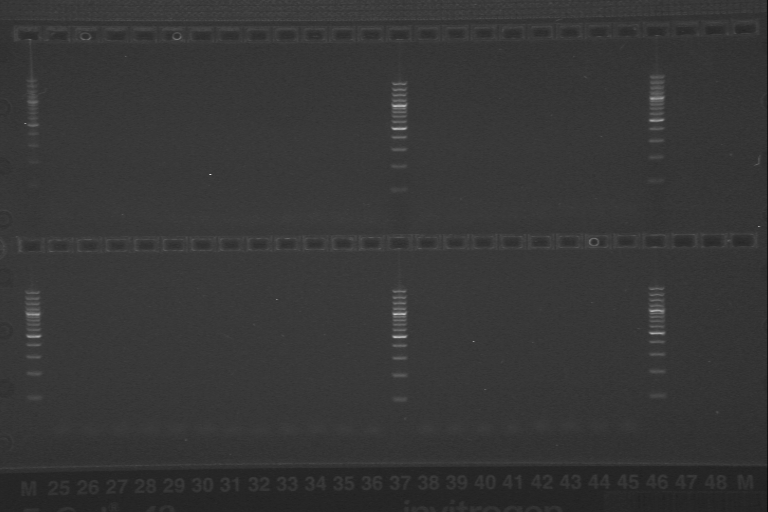


***Figure 3 - Gel 2****: Gel electrophoresis results of for the second gel. None of the negative samples show any bands. The top row contains N1 through N20 mixed with pair 2, and the bottom row contains N1 through N20 mixed with pair 3. None of the negative samples show any bands.*
